# Organizational commitment of emergency physician and its related factors: A national cross-sectional survey in China

**DOI:** 10.3389/fpubh.2022.936861

**Published:** 2022-07-25

**Authors:** Ke Peng, Xiaotong Han, Nan Jiang, Rongrong An, Chuanzhu Lv, Shijiao Yan

**Affiliations:** ^1^Department of Finance, Southampton Business School, University of Southampton, Southampton, United Kingdom; ^2^Hunan Provincial Key Laboratory of Emergency and Critical Care Metabolomics, Department of Emergency Medicine, Hunan Provincial Institute of Emergency Medicine, Hunan Provincial People's Hospital/The First Affiliated Hospital, Hunan Normal University, Changsha, China; ^3^Department of Social Medicine and Health Management, School of Public Health, Tongji Medical College, Huazhong University of Science and Technology, Wuhan, China; ^4^Department of Emergency Medicine, Sichuan Provincial People's Hospital, University of Electronic Science and Technology of China, Chengdu, China; ^5^Research Unit of Island Emergency Medicine, Chinese Academy of Medical Sciences (No. 2019RU013), Hainan Medical University, Haikou, China; ^6^Key Laboratory of Emergency and Trauma of Ministry of Education, Hainan Medical University, Haikou, China; ^7^School of Public Health, Hainan Medical University, Haikou, China; ^8^Key Laboratory of Emergency and Trauma of Ministry of Education, Hainan Medical University, Haikou, China

**Keywords:** emergency department, physicians, organizational commitment, workplace violence, organization structure

## Abstract

**Background:**

Organizational commitment is important for job performance and employee retention. However, studies on the level of organizational commitment and its related factors among emergency physicians in China are scarce. Therefore, this study aimed to identify the factors associated with organizational commitment among emergency physicians in China.

**Methods:**

A national cross-sectional study was conducted in 2018 among emergency physicians in China. Data were collected from 10,457 emergency physicians using a standard structured anonymous questionnaire, including demographic characteristics, organizational structure factors and work environment factors. A generalized linear model was used to explore the correlation between the independent variables and organizational commitment.

**Results:**

In this study, 55.3% of emergency physicians reported a moderate level of organizational commitment. The physicians who were male, younger than 40 years old, had a mid-level title and had a lower average monthly income were more likely to show lower organizational commitment levels. Conversely, the organizational commitment was higher among physicians who perceived that promotion is easy and the number of emergency physicians meet their daily work or had not experienced workplace violence in the last year.

**Conclusions:**

The study showed that organizational commitment among Chinese emergency physicians was moderate and related to gender, age, monthly income, frequency of daily visits, departmental promotion mechanism and workplace violent. Targeted interventions are needed to improve the organizational commitment of emergency physicians in a comprehensive way.

## Introduction

The high turnover rate of emergency physician has become a worldwide problem (1), which will exacerbate the shortage of human resources in the emergency department, leading to an inadequate supply of emergency medical services and affecting the quality of medical services (2). Previous studies indicate that organizational commitment has a significant impact on physicians' turnover intention (3, 4), organizational commitment is defined as a psychological state to his or her organization, including a strong desire to stay in the organization and a strong belief to achieve organizational goals (5, 6).

As with professions of teachers (7), military personnel (8) and administrators (9), organizational commitment is predictive of key outcomes among medical service providers, including long-term intention to stay in the workplace, productivity and responsibility. However, research shows that the level of organizational commitment of doctors is often lower than that of other social professions such as real estate and teaching staff (9). The decrease in physicians' organizational commitment leads to negative personal and organizational consequences, such as ineffective treatment, diminished patient trust, and high turnover rates (10–13). In addition, since the training of medical personnel is generally more extensive, time-consuming and expensive than other professions, it is particularly important to conduct organizational commitment studies for medical workers, especially in resource-starved emergency departments (11, 14).

Previous studies have shown that the organizational commitment of physician is related to demographic characteristics, job-related factors. For example, the Finnish study reported a significant relationship between organizational commitment level and doctors' personality (15), while the Chinese study on medical staff emphasized the positive relationship between organizational commitment, work experience and managers' trust (16, 17). The research on the staff of Iran medical center and Shanghai psychiatric department shows that there is a significant positive correlation between the ethical atmosphere and incentive system of the department and the level of organizational commitment of doctors (10, 18). The majority of previous research on factors related to physicians' organizational commitment have been conducted among physicians in neonatology, internal medicine and other departments (19), with less empirical investigations on emergency department physicians' organizational commitment. Only one study in Spain showed that emergency physicians' organizational commitment was moderate and related to physician competence and work experience (20). In addition, as compared to physicians in other departments, emergency physicians work in a highly stressful and fast-paced working environment. Therefore, emergency physicians are more likely to leave their existing professions (21), and emergency departments have a pressing need to lower physician turnover rates (11). In this context, the lack of studies on factors related to organizational commitment of emergency department physicians may hinder the development of interventions specific to emergency department characteristics.

The demand for emergency medical services in China has risen considerably in recent years, but there has been a severe turnover of emergency physicians. According to a study of medical resources in emergency departments in China, 55.18% of emergency physicians tended to leave their current positions (21). In the context of the mismatch between emergency demand and human resources among emergency department in China, it is very important to study factors related to the organizational commitment of emergency physicians to retain existing emergency physicians and improve the current situation of emergency services in China. To our knowledge, there are no studies on organizational commitment and its associated factors for emergency department physicians in China. Therefore, we conducted a nationwide survey on organizational commitment among emergency department physicians and explored its related factors from the aspects of demographic characteristics, organizational structure factors and work environment factors.

## Methods

### Ethics statement

This study was approved by the Medical Ethics Committee of Hainan Medical College (HYLL-2018-035). All participants were voluntary and anonymous, and all identifiable information in the questionnaire was kept confidential, and the content of the survey was used for scientific research only.

### Participants and data collection

We conducted a national cross-sectional survey of emergency medical resources from July to August 2018 under the coordination of the Medical Administration Bureau of the National Health Commission of the People's Republic of China. The survey used a standard structured questionnaire, and the collection and preservation of the data were handled by the Chinese online survey platform (platform name: Questionnaire Star, URL: https://www.wjx.cn). The link to the web-based questionnaire was posted on the emergency physicians' working platform, and emergency physicians were invited to participate anonymously in this online survey. To remind physicians to complete the survey, the electronic questionnaire was reposted to the survey platform every 7 days until the survey was completed. Participants who click on the link are first directed to an electronic informed consent statement page, which they must read and agree to before proceeding with the questionnaire. Each cell phone number could only be used once to prevent duplicate questionnaire submissions. The questionnaire could not be submitted if a participant did not answer all the questions. Hence there were no missing data in this study.

### Measurements

The questionnaire items covered demographic characteristics, organizational structure factors, work environment factors, and the organizational commitment of emergency physicians. The demographic characteristics included gender, age, education level, and self-rated health status. Organizational structure factors include professional title, monthly income level, job seniority, the average number of physician visits per day, perceived physician shortage and perceived promotion. Work environment factors include experienced verbal or physical violence in the past year.

Self-rated health status was evaluated by asking one question: “How do you think your health has been in the last six months?” was used to evaluate physician's self-rated health. The results were evaluated on 5-point Likert-type scale ranging from 1 (very good) to 5 (very poor). The physicians were divided into two groups based on their self-perceived health status scores: those who scored 1, 2, or 3 were classified as the good health group, while those who scored 4 or 5 were classified as the poor health group.

The physician shortage was evaluated by a question: “Do you think the current number of physicians in the emergency department meets the daily workload?” Results were assessed using a 5-point Likert-type scale (very satisfied = 1, satisfied = 2, fair = 3, unsatisfied = 4, very unsatisfied = 5). To simplify the analysis, the results were divided into two groups: those who scored 1, 2, or 3 thought the department had adequate physicians, while those who scored 4 or 5 thought the department had a physician shortage.

Promotion is assessed by a question: “Do you think it is easy to get promoted in the current section?” (“easy” and “not easy”).

Workplace violence encompasses both verbal and physical violence, and is asked using a separate question, “In the past year, have you experienced verbal (physical) violence at work?” (“No” and “Yes”).

Organizational commitment was measured according to the Organizational Commitment Scale developed by Mowday et al. (22–24). This scale has been used in several studies to describe physicians' perceived association and loyalty to the organization and has proven good reliability (23). The scale consists of six items, and the items were evaluated on a 5-point Likert-type scale (from 1 = strongly disagree to 5 = strongly agree). The average of the six question scores represents organizational commitment, with higher scores indicating higher levels of organizational commitment. Referring to the analysis in other studies (5, 25), the level of organizational commitment was classified into three levels: low level of organizational commitment (1.00–2.33), medium level of organizational commitment (2.34–3.66), and high level of organizational commitment (3.67–5.00). In this study, Cronbach's α is equal to 0.85, indicating adequate reliability.

### Statistical analysis

This study used Statistical Package for the Social Science (SPSS) version 23 (SPSS Inc., Chicago, IL, USA) to analyze the collected data. Descriptive analyses were conducted for physicians' demographic characteristics, organizational structure factors and work environment factors and organizational commitment. Continuous variables were presented as means and standard deviations, and categorical variables were presented as frequencies and percentages. *T*-test or analysis of variance (ANOVA) was conducted to compare the organizational commitment score of each group of physicians. When the homogeneity test of variance was significant, separate Satterthwaite t' test or Welch test were used. Multicollinearity of the independent variables was tested by calculating the Variance Inflation Factor (VIF) (max VIF = 2.083 and minimum = 1.051) (Supplementary Table S1). Due to the organizational commitment was not normally distributed, the relevant factors of organizational commitment of physicians in the emergency department were explored using generalized linear regression. All statistical tests were two-tailed, and a P-value < 0.05 was considered statistically significant.

## Results

### Participant characteristics

The characteristics of the participants are showed in Table 1. Among the 10,457 emergency physicians, most of them (73.0%) were male. The majority of the participants (84.4%) were married and nearly 70% obtained a bachelor's degree. More than half (63.4%) of the physicians reported good self-perceived physician health. The proportion of physicians reporting staff shortage was 73.3%. Moreover, about one-third (27.6%) of participants experienced physical violence and 81.8% of physicians experienced verbal violence in the past year.

**Table 1:** Descriptive statistics of the distribution of levels of organizational commitment among emergency physicians.

**Variables**	**Total** ***n*** **(%)**	**OC scores** **(mean** ±**SD)**	**Low OC** ***n*** **(%)**	**Medium OC** ***n*** **(%)**	**High OC** ***n*** **(%)**	**Statistical** **values (*****F*****/*****t*****)**	***P*** **-value**
**Total**	10,457 (100.0)	3.09 ± 0.76	1,860 (17.8)	5,784 (55.3)	2,813 (26.9)		
**Sex**						86.95	<0.001
Male	7,632 (73.0)	3.05 ± 0.78	1,498 (19.6)	4,212 (55.2)	1,922 (25.2)		
Female	2,825 (27.0)	3.20 ± 0.70	362 (12.8)	1,572 (55.6)	891 (31.5)		
**Age**						19.18	<0.001
<30	2,600 (24.9)	3.12 ± 0.76	417 (16.0)	1,452 (55.8)	731 (28.1)		
31–40	4,977 (47.6)	3.04 ± 0.76	971 (19.5)	2,798 (56.2)	1,208 (24.3)		
41–50	2,388 (22.8)	3.13 ± 0.76	409 (17.1)	1,281 (53.6)	698 (29.2)		
≥51	492 (4.7)	3.25 ± 0.75	63 (12.8)	253 (51.5)	176 (35.8)		
**Marit status**						0.13	0.908
Married	8,828 (84.4)	3.09 ± 0.76	1,569 (17.8)	4,890 (55.4)	2,369 (26.8)		
Unmarried/other	1,629 (15.6)	3.09 ± 0.78	291 (17.9)	894 (54.9)	444 (27.3)		
**Education level**						65.36	<0.001
Under bachelor	1,684 (16.1)	3.28 ± 0.76	203 (12.1)	849 (50.4)	632 (37.5)		
Bachelor	7,789 (74.5)	3.05 ± 0.75	1,490 (19.1)	4,379 (56.2)	1,920 (24.7)		
Master or higher	984 (9.4)	3.11 ± 0.75	167 (9.0)	556 (56.5)	261 (26.5)		
**Title**						44.12	<0.001
Junior or less	4,972 (47.5)	3.14 ± 0.76	793 (15.9)	2,710 (54.5)	1,469 (29.5)		
Intermediate	4,112 (39.3)	3.00 ± 0.76	856 (20.8)	2,317 (56.3)	939 (22.8)		
Senior	1,373 (13.1)	3.15 ± 0.73	211 (15.4)	757 (55.1)	405 (29.5)		
**Monthly income (CNY)**						4.15	0.006
< ¥4,000	3,862 (36.9)	3.07 ± 0.78	745 (19.3)	2,104 (54.5)	1,013 (26.2)		
¥4,001–6,000	3,562 (34.1)	3.08 ± 0.76	632 (17.7)	1,981 (55.6)	949 (26.6)		
¥6,001–8,000	1,904 (18.2)	3.11 ± 0.73	313 (16.4)	1,061 (55.7)	530 (27.8)		
≥¥8,001	1,129 (10.8)	3.15 ± 0.72	170 (15.1)	638 (56.5)	321 (28.4)		
**Work tenure (year)**						21.62	<0.001
<1	1,448 (13.8)	3.20 ± 0.73	199 (13.7)	781 (53.9)	468 (32.3)		
1–5	3,965 (37.9)	3.11 ± 0.76	658 (16.6)	2226 (56.1)	1,081 (27.3)		
6–10	2,458 (23.5)	3.02 ± 0.78	497 (20.2)	1391 (56.6)	570 (23.2)		
≥11	2,586 (24.7)	3.06 ± 0.76	506 (19.6)	1386 (53.6)	694 (26.8)		
**The number of patients seen by the physicians (per day)**						26.48	<0.001
1–10	4,333 (41.4)	3.16 ± 0.76	700 (16.2)	2,324 (53.6)	1,309 (30.2)		
11–20	2,202 (21.1)	3.10 ± 0.77	389 (17.7)	1,211 (55.0)	602 (27.3)		
21–30	1,547 (14.8)	3.06 ± 0.75	284 (18.4)	867 (56.0)	396 (25.6)		
≥31	2,375 (22.7)	2.99 ± 0.76	487 (20.5)	1,382 (58.2)	506 (21.3)		
**Self-perceived easy promotion**						150.97	<0.001
Yes	2,253 (21.5)	3.26 ± 0.72	225 (11.3)	1,214 (53.9)	784 (34.8)		
No	8,204 (78.5)	3.04 ± 0.76	1,605 (19.6)	4,570 (55.7)	2,029 (24.7)		
**Self-perceived sufficient physicians**						685.206	<0.001
Yes	2,790 (26.7)	3.40 ± 0.68	206 (7.4)	1,422 (51.0)	1,162 (41.6)		
No	7,667 (73.3)	2.98 ± 0.75	1,654 (21.6)	4,362 (56.9)	1,651 (21.5)		
**Self-perceived health condition**						1,092.66	<0.001
Good	6,629 (63.4)	3.27 ± 0.70	715 (10.8)	3,668 (55.3)	2,246 (33.9)		
Not good	3,828 (36.6)	2.78 ± 0.77	1,145 (29.9)	2,116 (55.3)	567 (14.8)		
**Experienced verbal violence in the past year**						591.75	<0.001
No	1,902 (18.2)	3.46 ± 0.70	145 (7.6)	876 (46.1)	881 (46.3)		
Yes	8,555 (81.8)	3.01 ± 0.75	1,715 (20.0)	4,908 (57.4)	1,932 (22.6)		
**Experienced physical violence in the past year**						467.58	<0.001
No	7,568 (72.4)	3.19 ± 0.72	1,075 (14.2)	4,158 (54.9)	2,335 (30.9)		
Yes	2,889 (27.6)	2.84 ± 0.76	785 (27.2)	1,626 (56.3)	478 (16.5)		

OC, organizational commitment.

### Organizational commitment of emergency physicians

Table 1 demonstrated the distribution of participants' organizational commitment. The mean level of organizational commitment of emergency physicians in China was 3.09 ± 0.76, which is a moderate level of organizational commitment. In this survey, 17.8% of physicians were at a low level of organizational commitment, 55.3% of participants were at a medium level of organizational commitment and 26.9% of respondents were at a high level of organizational commitment. The results of univariate analysis revealed that the organizational commitment level of emergency physicians differed significantly between groups in the following characteristics: gender, age, education level, self-perceived health, monthly income, job seniority, the average number of physician visits per day, perceived physician shortage, perceived promotion, and workplace violence in the past year (*p* < 0.05).

### Generalized linear regression analysis results

The results of the generalized linear model analysis are shown in Table 2. Regarding individual factors, male emergency physicians were more likely to had low levels of organizational commitment compared to female physicians (*p* < 0.001, β = –0.08). Physicians aged less than 30 years old (*p* < 0.001, β = −0.14) and aged 31-40 years old (*p* < 0.05, β = –0.08), with a bachelor's degree or below (*p* < 0.001, β = 0.14)and with good self-perceived physical health (*p* < 0.001, β = 0.33) had higher levels of perceived organizational commitment. Regarding organization structure factors, emergency physicians with a monthly income of less than ¥6,000performed lower organizational commitment scores than those with a monthly income of more than ¥8,000(*p* < 0.001, β = –0.12). And physicians in the emergency department with more than 31 visits per day had a lower organizational commitment level than those with fewer than 10 visits per day (*p* < 0.05, β = 0.05). Additionally, the physicians who had self-perceived ease of promotion (*p* < 0.001, β = 0.06) and were never exposed to verbal violence (*p* < 0.001, β = 0.20) and physical violence (*p* < 0.001, β = 0.16) demonstrated higher levels of perceived organizational commitment.

**Table 2:** Correlation between independent predictor variables and level of organizational commitment.

**Variables**	**B**	**SE**	**Wald**	***P-*** **value**	**OR**
**Sex (Ref: Female)**					
Male	−0.08	0.02	24.78	<0.001	0.92
**Age (Ref: ≥51)**					
≤ 30	−0.14	0.04	12.06	<0.001	0.87
31–40	−0.08	0.04	5.14	0.023	0.92
41–50	−0.01	0.03	0.28	0.596	0.98
**Marital status (Ref: Unmarried/others)**					
Married	−0.06	0.02	8.45	0.004	0.94
**Education level (Ref: Master degree or above)**					
Less than bachelor	0.14	0.03	14.34	<0.001	1.15
Bachelor degree	0.01	0.02	0.01	0.697	1.01
**Title (Ref: Senior)**					
Junior or less	0.04	0.03	1.84	0.176	1.04
Intermediate	−0.07	0.02	8.83	0.003	0.93
**Monthly income (Ref: ≥¥8,001)**					
≤ ¥4,000	−0.12	0.03	22.39	<0.001	0.89
¥4,001–6,000	−0.06	0.02	5.51	0.019	0.94
¥6,001–8,000	−0.02	0.03	0.35	0.556	0.99
**Work tenure, year (Ref: ≥11)**					
<1	0.04	0.03	2.11	0.147	1.04
1–5	0.05	0.02	5.14	0.023	1.05
6–10	0.01	0.02	0.06	0.815	1.01
**The number of patients seen by the physicians (Ref: ≥31)**					
≤ 10	0.05	0.02	6.57	0.01	1.05
11–20	0.04	0.02	4.15	0.042	1.04
21–30	0.03	0.02	2.08	0.149	1.03
**Self-perceived easy promotion (Ref: No)**					
Yes	0.06	0.02	10.61	0.001	1.06
**Self-perceived sufficient physicians (Ref: No)**					
Yes	0.26	0.02	253.18	<0.001	1.30
**Self-perceived physical health (Ref: Bad)**					
Good	0.33	0.02	481.05	<0.001	1.39
**Experienced verbal violence in the past year (Ref: Yes)**					
No	0.20	0.019	135.65	<0.001	1.25
**Experienced physical violence in the past year (Ref: Yes)**					
No	0.16	0.02	98.38	<0.001	1.17

Ref, Reference.

## Discussion

Emergency physicians play a critical role as an important human link in the healthcare delivery system. Therefore, study on professional value of emergency physicians is important.

The results show that the average organizational commitment level of emergency physicians in China is consistent with that of psychiatrists and community health workers in China, which is at a medium level. However, the organizational commitment score of Chinese emergency doctors was lower than that of medical staff in other departments in China (17, 26, 27). The reason may be related to that emergency department physicians are exposed to a more complex work environments, heavier workload and greater mental pressure than physicians in other departments (11). In addition, probably due to differences in the stage of development of emergency medical service systems, socioeconomic conditions, and perceptions of organizational commitment among countries (11), the organizational commitment of emergency physicians in China was lower than that of physicians in the USA (3.3) (28), Emergency Medical Technicians in Iran (3.51) (29) and emergency physicians in Spain (3.8) (30).

There was a statistically significant difference in organizational commitment between males and females among physicians in emergency department. Male physicians were more likely to have lower levels of organizational commitment compared to female physicians, which is consistent with previous surveys of Chinese specialist physicians (18). The main reason for the result was that, in the context of traditional Chinese culture, males tend to have a stronger need for job achievement and career development expectations. Therefore, when physicians are dissatisfied with their current organization, males are more likely to seek jobs with better pay and better job opportunities (21, 31). Similarly, emergency physicians with higher education had lower levels of organizational commitment, possibly because they had more job opportunities in the current health care system and a stronger competitive advantage, making it easier for them to leave their current positions for higher-paying jobs (32). In this case, it is necessary for hospital managers to increased structural empowerment to define new roles for emergency physicians based on their characteristics, and attempts can be made to attract and retain highly educated personnel with salary increases and invitations to join the management team (33). In addition, physicians who had good health status were more likely to have a high organizational commitment. This conclusion suggests that to improve emergency physicians' organizational commitment, hospital administrators should pay attention to their physical health and safeguard their health through frequent health checks and other measures (34).

Organizational structure is critical to improving the level of organizational commitment. First, emergency physicians with higher monthly incomes were more likely to perceive high levels of organizational commitment, which has been described in previous studies conducted among Korean doctors and Chinese medical staff (18, 35). The income level is a direct reward for the effort, and the high income makes emergency physicians feel that their support for the organization is worthy. Similarly, those emergency physicians with higher professional titles believed their efforts had achieved a higher sense of professional achievement and self-esteem, so they had a higher sense of organizational commitment. Moreover, This study obtained an interesting finding that physicians who perceived a manpower shortage would be lower organizational commitment. The possible explanation is that the shortage of physicians in the department means a greater workload for emergency physician on duty, increasing physician stress and making it easier to feel fatigued at work (21, 35). There was no such report in existing studies on the correlation between self-perceived shortage of workforce and organizational commitment among physicians. Furthermore, emergency physicians who reported easy advancement were more likely to have high levels of organizational commitment. The possible explanation is that a rational promotion system increases physicians' continuous commitment to the organization, increase the well-being of doctors institutionally and meet the professional needs. And similar results have been reported in Korea and China to explain the phenomenon that Promotion system affects organizational commitment (17, 18, 36). As a result, organizations with a competent promotion structure and adequate human resource staffing within the department may give better growth opportunities and a more compassionate load to physicians, resulting in a positive effect on organizational commitment (37, 38).

Furthermore, this study showed that physicians who had not exposed to verbal or physical workplace violence had higher levels of organizational commitment than those who had suffered workplace violence. This might be because workplace violence not only harms the physical health of physicians, but it also causes negative emotions such as anxiety and resistance, as well as post-traumatic stress disorder. These psychological and emotional injuries burden physician's daily work and lives and reduce their sense of identity with their work and organization. Studies in Finland and China also found that workplace violence had an inverse effect on healthcare workers' organizational identity (39, 40). Different from other departments, those patients admitted to the emergency department with severe illness or injury are more emotionally unstable, and there is not enough time for communication due to the urgency of the incident, so emergency physicians are more likely to suffer from workplace violence (20, 41). A survey of nurses in Turkish hospitals also found that a good nursing work environment has a positive effect on nurses' organizational commitment (42). Therefore, hospital administrators should ensure a safe work environment for emergency physicians by developing reasonable protection policies. Strengthening physicians' interpersonal communication skills to reduce the impact of workplace violence on organizational commitment.

### Strengths and limitations

The strength of this study was that it was the first time to report the organizational commitment level and related factors of emergency physicians in China. In addition, the participants were distributed across multiple provinces and cities, and the large sample was able to enhance the statistical power of the organizational commitment status of emergency physicians. so the results of this study have fairly representative.

However, there are some limitations to the findings. Firstly, most of the information relied on respondents' self-reported questionnaires, making it difficult to completely avoid recall bias. Secondly, as this study was a cross-sectional design, and causal relationships cannot necessarily be made between the independent variables and organizational commitment. Further cohort studies with larger samples are required.

## Conclusion

The organizational commitment among Chinese emergency physicians was at a moderate and related to organizational structural factors (workload, self-perceived physician satisfaction, promotion mechanisms) and work environment factors (workplace violence). Given that emergency physicians play an important role in the emergency medical workforce, while the shortage of emergency physicians is persistent. The results suggest that hospital administrators should improve promotion mechanisms, rationally allocate department human resources, take measures to reduce workplace violence, ensure the safe working environment in emergency rooms, and promote high-quality emergency medical care.

## Data availability statement

The raw data supporting the conclusions of this article will be made available by the authors, without undue reservation.

## Ethics statement

This study was approved by the Medical Ethics Committee of Hainan Medical College (HYLL-2018-035). All participants were voluntary and anonymous, all identifiable information in the questionnaire was kept confidential, and the content of the survey was used for scientific research only.

## Author contributions

KP, CL, and SY designed this study and collected the data. KP and RA completed data analysis and drafted the main manuscript text. NJ, CL, and SY provided the critical revision of the manuscript and supervision all the processes of the work. XH was responsible for the conception, design, and writing of the manuscript. All authors reviewed and approved the manuscript.

## Funding

This study was supported by the Major Science and Technology Project of the Hainan Provincial Department of Science and Technology (ZDKJ202004) and the Key Research and Development Program of the Hainan Provincial Department of Science and Technology (ZDYF2020112).

## Conflict of interest

The authors declare that the research was conducted in the absence of any commercial or financial relationships that could be construed as a potential conflict of interest.

## Publisher's note

All claims expressed in this article are solely those of the authors and do not necessarily represent those of their affiliated organizations, or those of the publisher, the editors and the reviewers. Any product that may be evaluated in this article, or claim that may be made by its manufacturer, is not guaranteed or endorsed by the publisher.
