# Supplementary material for: Organizational commitment of emergency physician and its related factors: A national cross-sectional survey in China
Source: Front Public Health. 2022 Jul 25;10:936861. doi: 10.3389/fpubh.2022.936861 (PMC9357984; doi:10.3389/fpubh.2022.936861)
Supplement: Supplementary file 1 [file Table_1.DOCX]

Table S1. The Multicollinearity test results

| **Variables** | **Tolerance** | **Variance**  **inflation factor** |
| --- | --- | --- |
| **Gender** | 0.95 | 1.05 |
| **Age** | 0.48 | 2.08 |
| **Marital status** | 0.87 | 1.15 |
| **Education level** | 0.84 | 1.19 |
| **Title** | 0.51 | 1.95 |
| **Average monthly income** | 0.84 | 1.19 |
| **Work tenure** | 0.64 | 1.57 |
| **The number of patients seen by the physicians** | 0.91 | 1.09 |
| **Self-perceived easy promotion** | 0.92 | 1.09 |
| **Self-perceived sufficient physicians** | 0.82 | 1.13 |
| **Self-perceived health condition** | 0.88 | 1.14 |
| **Experienced verbal violence in the past year** | 0.86 | 1.17 |
| **Experienced physical violence in the past year** | 0.88 | 1.14 |
